# Extended home visits can provide multidimensional adapted professional support for parents – an intervention study

**DOI:** 10.1017/S1463423623000336

**Published:** 2023-07-05

**Authors:** Margaretha Larsson, Caroline Bäckström, Rebecca Larsson, Sara Gahm, Marie Wilhsson

**Affiliations:** 1 School of Health Sciences, University of Skövde, Skövde, Sweden; 2 Faculty of Caring Science, Work Life and Social Welfare, University of Borås, Borås, Sweden; 3 Primary health centre Bra liv, Bankeryd, Sweden; 4 Primary health center Norrmalm, Skövde, Sweden

**Keywords:** child health nurse, collaboration, family support, family supporter, midwife, parents, patient-centred care, professional support, qualitative research

## Abstract

**Aim:**

The aim of this study was to explore healthcare professionals’ experiences of working with extended home visits for parents.

**Background:**

It is essential to identify parents, both expectant and with a newborn child, who need support in their parenting abilities at an early stage because children’s health and well-being are affected by their home environment as well as by their parents’ health and social relationships. Home visits represent a cost-effective way of identifying and supporting families with a newborn. Further research is needed to explore healthcare professionals’ experiences working with extended home visits for parents.

**Methods:**

This was a qualitative interview study focusing on an intervention introduced in the *Enhanced Parenting—Extended Home Visits* project in Sweden. Data were collected via 13 semi-structured interviews with healthcare professionals who provide the intervention in antenatal care (midwives) and child health care (CHC nurses and family supporters), and a qualitative content analysis was performed.

**Findings:**

Data analysis resulted in one theme and four categories. The theme – to provide multidimensional adapted professional support, – and the four categories – strengthened collaboration between professionals enriches their work. Home visits provide time for conversation, which promotes continuity of care and relationships with parents; being humble guests in parents’ homes provides insight; and home visits provide the opportunity to strengthen parenting and participation in the family centre. The goals of the *Enhanced Parenting—Extended Home Visits* project were to strengthen parents’ confidence in their parenting abilities and to build trusting relationships with healthcare professionals. The conclusion of this study, from the participants’ perspective, is that these goals can be achieved with the intervention.

**Implications for Practice:**

Extended home visits seem to help healthcare professionals provide collaborative, multi-professional support for parents, both expectant and with a newborn child, with unique support needs.

## Introduction

International research has shown that when child health care (CHC) nurses, in collaboration with family supporters (within social service), offer parents more home visits during their children’s first year of life, their parenting abilities are strengthened, which has positive effects on their children’s health (Aronen and Arajarvi, 2000; Olds *et al.*, 2007; Barboza *et al.*, 2018). Extended home visits can develop parents’ knowledge, skills and motivation for positive parenting and strengthen the health and functioning of the family, which supports children during both childhood and adolescence (Aronen and Arajarvi, 2000). The Commission on Social Determinants of Health (CSDH, 2008) was established by the World Health Organization (WHO) in 2005 to promote health equity. Especially, it emphasises promoting a good start in life to achieve health equity (Irwin *et al.*, 2007). The Health and Medical Care Board in Skaraborg, southeast Sweden, implemented a strategic plan for public health work in 2010, which particularly highlighted support for vulnerable groups, for example, parents, both expectant and with a newborn child, in socio-economically disadvantaged areas. Focus was placed on developing collaboration between professionals working in antenatal care, CHC and family support, which is a part of the Swedish social service provided at family centres. In Sweden, an intervention with a home visit programme was developed in collaboration with antenatal care and CHC and with family support and family centres. The main goal was to offer professional support by healthcare professionals representing various professions (i.e., midwives, CHC nurses and family supporters). This allowed for the different healthcare professionals to complement each other’s professional knowledge in meetings with expectant parents and parents with a newborn child.

## Background

Internationally, professionals working in antenatal care and CHC have a responsibility to provide support for parents. In terms of realising the rights and well-being of women, men and children, access to high-quality family planning services is fundamental (WHO, 2018). Professional support is offered to expectant parents within antenatal care and to parents with a newborn child within CHC. In Sweden, the antenatal care and CHC service has been shown to serve as an important form of professional support, one aimed to promote children’s health and psychosocial development and strengthening parents in their parental role (Swedish National Board of Health and Welfare, 2014). Globally, antenatal care and CHC should be free of charge and based on the Convention on the Rights of the Child (CRC) with a child perspective and in the child’s best interest (UNICEF, 1989; SFS, 2018: 1197).

Previous review articles have shown that by developing a trusting relationship with parents, professional support can both promote parental role construction and provide guidance to parents, which can in turn more effectively facilitate the transition to parenthood and create a safe environment for the children (Reticena *et al*., 2019). Coyne *et al*. (2013) maintained that it is important to create space for conversation between professionals and families to build confidence and trust in the family. In a discourse paper, professional support in child-bearing is described and problematised from several aspects, and its benefits, such as positive experiences for parents, longer breast-feeding duration and improved mother–infant interaction, are highlighted, as well as the importance of meeting parents’ individual support needs (Ekström-Bergström *et al*., 2022). Moreover, professional support helps parents feel more confident in their parenting skills and cultivates general well-being (Stubbs and Achat, 2016). It is valuable for professionals to bear in mind that parents have individual support needs, such as in cases of mental or social illness, which make it challenging for the parents to fulfil their parental role (Reticena *et al.*, 2019).

Globally, expectant parents are usually cared for within antenatal care, although the types of healthcare professionals responsible for providing antenatal care and family planning services vary (WHO, 2018). Midwives in Swedish antenatal units are independently responsible for managing healthy pregnancies, offering expectant parents six to nine antenatal visits; these visits could be seen as health check-ups intend to detect any pregnancy-related complications (Banke *et al.*, 2008).

CHC promotes child health by offering health examinations, guidance and vaccinations for all children (Swedish National Board of Health and Welfare, 2014). In Sweden, CHC is staffed by professionals, such as CHC nurses, who provide families with access to physicians, psychologists, dieticians, social workers and speech therapists (Tell *et al.*, 2018). In Sweden, similar to many other countries, CHC nurses are specialist nurses. They are certified and have a postgraduate nurse education with a master’s degree. CHC nurses first meet parents at the birth of their child, after which the family receives, on average, 13 visits throughout the first five years of their child’s life. Of these visits, two are home visits (the initial when the child is two weeks old and the second occurs when the child is eight months old), whereas the remaining visits take place at a CHC centre.

In Sweden, similar to many other countries (Kekkonen *et al.*, 2012), there are also family centres that offer professional support coordinated at the central level in the municipality. These centres, which are focused on promotion, cover antenatal health, CHC, open preschool and social services (Swedish National Board of Health and Welfare, 2014). The organisation of family centres is based on a national strategy for community assistance and is intended to support parents in their parenthood (SOU, 2008: 131). Open preschools are a unique form of preschool because children attend them with their parents when parents choose to do so, it is free of charge, and no prior registration is required. The Swedish social service is a municipal administration ordered by the municipality’s social committee and regulated by law (SFS, 2001: 453). Family supporters have a bachelor’s degree in social work. Working in social services entails responsibility for protecting children from growing up in poor conditions through outreach activities. In Sweden, the goal is to create societal conditions for good health throughout the population (SOU, 2008: 131).

Becoming a parent can be described as a radical change in life that includes physiological, psychological and social changes (Cowan and Cowan, 2000). Life takes a different direction for new parents when their child is born (SOU, 2008: 131). Experiences of health are related to people’s living habits and living conditions as well as their socio-economic position (WHO, 2014). There is a social gradient in health, meaning that a low socio-economic position, a low level of education and a lack of employment can be risk factors for ill health within families. Equity in health is central to establishing a good start in life for children (CSDH, 2008). To achieve greater equity and equality in health, the WHO (CSDH, 2008) has recommended that universal interventions should be expanded, concentrating on groups that have the greatest needs and that use existing care less. The Swedish population is considered to have good overall health, but differences still exist in the population, mainly because of socio-economic factors (SOU, 2017: 47). It is essential to identify more vulnerable families at an early stage because children’s health and well-being are affected by their home environment as well as by their parents’ health and social relationships (Welch *et al*., 2001).

Research has shown that the quality of parental relationships decreases after the birth of their first child, which indicates that many new parents experience parenthood as a sensitive period in their lives (Bäckström *et al.*, 2018). It is thus important to support parents during their transition to parenthood using different approaches and to be sensitive to their unique needs (Gilmer *et al.*, 2016). Changing roles can lead to increased stress in the family and conflicts in relationships. Mangrio and Persson (2017) showed that parents in Sweden, who were born outside the EU, were grateful for the Swedish CHC and home visits because not all of them received this type of help in their former home countries. Home visits represent a cost-effective way of identifying and supporting vulnerable families (Olds *et al.*, 2007; Barboza *et al.*, 2018). Previous research has shown that during extended home visits, children and their families behave more naturally, which allows the children to behave more characteristically and gives parents a feeling of enhanced security that can facilitate conversations with professionals (anonymous date in manuscript). Another study described how extended home visits can strengthen relationships between parents and children, children and CHC nurses and parents and CHC nurses (Stubbs and Achat, 2016). For professionals, home visits provide opportunities to pay attention to the environment in which a family lives, making it possible for them to increase their understanding of the family and thereby better meet the family’s unique needs (Leirbakk *et al.*, 2018). With the unique family in focus, professionals can promote both the family’s health and the children’s upbringing (Larsson *et al.*, 2020). Extended home visits to socially disadvantaged parents with a newborn child have been shown to reduce children’s behavioural problems, the frequency of accidental harm (Aronen and Arajarvi, 2000) and the presence of risks in the home (Hahn *et al.*, 2003). However, how professionals experience working within the intervention, including these extended home visits, remains an under-explored issue. Hence, this study aimed to explore professionals’ experiences working with extended home visits to parents. In the methods section, the term ‘extended home visits’ will be further explained.

## Methods

### Design

The present study stemmed from an intervention initiated in the *Enhanced Parenting—Extended Home Visits* project (anonymous date in manuscript). The intervention included professional home visits to parents during pregnancy and the first 15 months of parenthood, as explained more clearly below. The professional home visits were added to standard care, which means an ‘enhanced’ home visiting service, that is, different from standard care. Therefore, professional home visits are termed ‘extended home visits’ in both the project and the current study. The qualitative study uses an inductive approach to gain a deeper understanding of the healthcare professionals’ experiences working with extended home visits to families during pregnancy and with a newborn child in Sweden. Data were collected through individual semi-structured interviews, and a content analysis, as described by Graneheim and Lundman (2004), was performed.

### The intervention

Based on the Swedish national strategy for community support and assistance to parents in their parenting (SOU, 2008: 131), the intervention was developed to expand efforts to offer professional support to expectant and new parents. The intervention included professional home visits – that is, ‘extended home visits’ to expectant and new parents. These visits are provided by healthcare professionals in public antenatal care (midwives), CHC (CHC nurses) and social services (family supporters). The intention was that the same CHC nurses and family supporters would visit the same families, to achieve continuity in care. The intervention provides professional home visits additional to standard care, which could involve (1) an extra home visit not included in standard care (gestational week 34), (2) a family supporter participating in a standard home visit provided by a CHC nurse (at two weeks and eight months after birth) and (3) a home visit instead of a standard visit at the CHC unit (at four and 15 months after birth). The difference between the intervention and standard care is clarified in Table 1.

**Table 1.** Overview of the intervention consisting of extended home visits for parents during pregnancy and the first 15 months of parenthood

Extended home visit	Time point	Professionals responsible for the extended home visit	Issues brought up during extended home visit	Difference from standard care
1. To become a parent	Gestational week 34	Midwife working within antenatal care and family supporter working within social services	Relation to and support from partner; social network (family, friends and significant others); the expected child and the child’s needs; first time at home after birth; parenthood; emotional changes/reactions; breastfeeding; skin-to-skin contact	For the intervention, this home visit is conducted by a midwife and a family supporter, in addition to standard care
2. To meet and receive the child	Two weeks after birth	Child health nurse working within child health care (CHC) and family supporter working within social services	The child’s sleeping position; safety of the child; the child’s eating; how to comfort a sad child; the parents’ health and well-being; alcohol and tobacco use; the parental–couple relationship; violence in close relations; the parents’ social network; child health service and professional caregivers available for the family	In standard care, parents are visited at home two weeks after birth; for the intervention, a family supporter working for social services is present during the standard care home visit two weeks after birth
3. To be together	Four months after birth	Child health nurse working within CHC and family supporter working within social services	The child’s well-being; routines for the child’s eating and sleeping and language development (e.g., babbling and laughing); safety of the child; infections and self-care; the library and children’s books; the parents’ health and well-being; alcohol and tobacco use; the parental–couple relationship; violence in close relations; social media; CHC and professional caregivers available for the family	For the intervention, parents were provided with a home visit instead of visiting a CHC unit, which is included in standard care
4. To lead and follow	Eight months after birth	Child health nurse working within CHC and family supporter working within social services	The child’s language development; daily routines and feelings of security; safety of the child; infections and self-care; dental care; kindergarten/preschool; parental leave; the parents’ health and well-being; violence in close relations	In standard care, parents are provided with a home visit eight months after birth; for the intervention, a family supporter working for social services is present during the home visit eight months after birth
5. To be a family	Fifteen months after birth	Child health nurse working within CHC and family supporter working within social services	The child’s eating and sleeping routines; the child’s communication, language development and digital screen time; what the child likes to do; infections and self-care; the parental–couple relationship; parents’ time for own interests; violence in close relations	For the intervention, parents are provided with a home visit instead of visiting a CHC unit, which is included in standard care

The intervention was provided in two regions in southwestern Sweden. The reasons for choosing these two regions were as follows: (1) they generally include parents from a lower socio-economic status, often with a migrant background, who have greater need for professional support, and (2) they have well-functioning, cooperating family centres. The intervention aimed to strengthen the self-confidence of parents and to promote their trust in antenatal care and CHC, as well as social services. Furthermore, the intervention sought to promote equality and participation among parents and to identify families with additional professional support needs at an early stage (Bäckström *et al.,* 2021).

For the intervention, the recruitment process lasted from May 2018 to May 2019. The inclusion criteria were parents who lived in the two regions chosen for the intervention and who were expecting their first child in Sweden (i.e., parents with a migrant background could have had a previous child born in another country). Parents were recruited between gestational weeks 29 and 32 by midwives at antenatal units. In total, 50 parents agreed to participate in the intervention, which took place from 2018 to 2020. Healthcare professionals who worked at the antenatal and CHC units and in social services in the two regions were informed about the *Enhanced Parenting—Extended Home Visits* project, the intervention and their specific roles in the intervention. In total, the intervention included five home visits between gestational week 34 and the first 15 months after birth. Each specific home visit is described in Table 1, which was inspired by a table in a previous publication describing parents’ experiences with the intervention (anonymous date in manuscript). Parents who declined to participate in the intervention were offered standard care. In Table 1, both the intervention and standard care are described.

### Settings and participants

The current study was implemented in the two regions in southwestern Sweden where the intervention took place. There are approximately 15,000 residents in both regions. Purposive sampling was used to select study participants with knowledge of the topics of the study. The inclusion criteria were as follows: professionals who had worked in the intervention initiated in the *Enhanced Parenting—Extended Home Visits* project. The heads of the public antenatal, CHC and social services in the regions granted permission for the study. Thereafter, information and requests to participate in the study were sent via email to midwives at the antenatal services, nurses at the CHC services and family supporters at the social service centre who met the inclusion criteria. All professionals (*n* = 13) who provided the intervention in the *Enhanced Parenting—Extended Home Visits* project were asked to participate in the current study, and all agreed to participate and contacted the researchers. Then, written information was provided before an interview was scheduled. The participants comprised 13 professionals, and their distribution was as follows: four midwives, six CHC nurses and three family supporters. The participants varied in age from 32 to 65 years and represented a broad variety of professionals with diverse experiences providing the intervention (extended home visits) to parents.

### Data collection

Data were collected via semi-structured individual interviews. The interview guide was developed by researchers with broad experience in qualitative methods and consisted of an initial general question: *How have you experienced working with extended home visits in the Enhanced Parenting—Extended Home Visits project?* Examples of follow-up questions are as follows: *Could you describe if and how the extended home visits affected your working day? How did you experience the response from the parents during the home visits? Do you experience the relationship between the professionals and parents to be affected by the home visits?* To encourage the participants to broaden their narratives, follow-up questions were used, such as the following: *Could you describe this further?* and *Could you give an example?* The participants were free to choose the time and place of the interviews. In total, 13 interviews were conducted: 10 were face-to-face interviews held at the interviewees’ workplace and three were held via telephone. The interviews were conducted by two MSc students (SG and RL) between January 2020 and February 2020 and lasted between 20 and 40 min; the interviews were recorded using an electronic device and then transcribed verbatim. Along with the interviews, a senior researcher (ML) supervised the MSc students to ensure that the interviews achieved satisfying quality. For example, the senior researcher read the anonymised transcripts and commented on the MSc students’ interview technique.

### Data analysis

Graneheim and Lundman’s (2004) description of qualitative content analysis was used as a guide when conducting the data analysis. Three of the authors (SG, RL and ML) read the transcribed interviews several times to gain an understanding of the overall content. Statements (units of meaning) that described the healthcare professionals’ experiences working with extended home visits were identified and condensed into comprehensive units. Then, a grouping process took place in which comprehensive units with similar meanings were grouped into codes. Groups of codes with similar meanings were gathered to form categories. In total, one theme and four categories arose during the analysis process. During the analysis, we iterated between the smaller details of the participants’ descriptions and the entirety of the data material. All authors participated in repeated discussions of the comprehensive units, codes, categories and theme. The theme describes the core meaning of the professionals’ experiences working with extended home visits. The four categories in the results section are illustrated with quotes from the interviews. The consolidated criteria for reporting qualitative research (COREQ) checklist was used to ensure quality (Tong *et al*., 2007; see Appendix 1).

### Ethical considerations

The current study complied with both the Declaration of Helsinki (World Medical Association, 2013) and Swedish law and was approved by the Regional Ethical Review Board in Gothenburg (Dnr, 2019: 03906). All participants were given both verbal and written information about the study before giving their consent to participate. Moreover, all participants had the opportunity to withdraw their participation at any time without specifying a reason and were guaranteed that their data would be treated confidentially. The results are presented with excerpts from the participants’ interviews; however, these excerpts are presented in an anonymous form to ensure that the identity of each participant is protected. Participants’ identities were handled confidentially, and the data material was stored in ways that only allowed access by the researchers.

## Results

### Theme: to provide multidimensional adapted professional support

The extended home visits implemented in collaboration with professionals with different knowledge bases serve as a foundation for providing parents with multidimensional adapted professional support. The strengthened collaboration between professionals was described as enriching when the participants gained an understanding of each other’s work and developed their working methods, a prerequisite for which was being flexible with their time when coordinating extended home visits. The participants experienced extended home visits as meaningful in that the visits provided time for conversation, which promoted continuity of care and trusting relationships with parents. The participants described that being guests with a humble attitude provided insights into families’ homes, which in turn permitted conversations focusing on both children’s and parents’ needs and situations. During extended home visits, the participants strove to involve both parents to promote equal parenting. Initiating extended home visits during pregnancy and continuing until the children reached 15 months of age entailed opportunities to provide professional support at an early stage and to promote parents’ participation in family centres’ activities.

### Strengthened collaboration between professionals enriches their work

The new way of working with interprofessional collaboration has contributed to building relationships with colleagues and the project’s other professionals. Collaboration between the professionals was shown to be enriched via the provision of a better understanding of each other’s work, and this collaboration, in turn, facilitated contact with one another, for example, discuss families. Some participants expressed that to further support the collaboration, all of the professionals could be gathered at nearby premises. Midwives and CHC nurses experienced that collaboration with family supporters led to greater accessibility to social services. Collaboration was described by one CHC nurse as follows:

We have met in groups of all professionals, which has given a strengthened relationship with mainly family supporters […]. We have made contacts; it is easy to call them to discuss and ask for advice. Also, working in a team […] with family supporters and maternity care, you get insight into their occupation. (Int 5 X)

Implementing extended home visits presupposes that the professionals are sufficiently flexible to be able to coordinate the visits. The participants pointed out that the collaboration required flexibility when planning a schedule for two people with different professionals in the project, and at some visits, an interpreter. They expressed difficulties in coordinating times that suited everyone, particularly for those visits involving newborns (at two weeks after birth) and those occurring during the holiday period (e.g., at Christmas time), but the strengthened collaboration increased their flexibility. The professionals also needed to be flexible when contacting the parents to book home visits. The home visits involving children who were 15 months old were sometimes difficult to book, which according to the CHC nurses was due to parents returning to work and the children starting preschool. The participants described their first experience of carrying out an extended home visit in this project as hectic, even though they had support from management and sufficient time was provided by their employers. The new way of working initially required more energy before practical things (e.g., scheme for booking home visits) and new routines could be established:

To find times, we can easily pick up the phone and talk to each other. (Int 3 X)

The participants experienced enriched collaboration as they learned from each other and developed their working practice. For example, in conversations with parents, the various professionals offered advice based on their knowledge; as one participant expressed, ‘It’s a bit like intertwining each other’s work’ (Int 5 X). Being able to participate in the project made participants feel more valued, although working independently and thus representing their profession on their own made them more vulnerable, as it required them to live up to the demands and expectations of their profession. The family supporter described that it was especially enjoyable to make the first home visit with the midwife when the focus was placed on the parents’ situation before the child was born. For the midwives, this was a completely new way of working; they had not previously made any home visits and described them as exciting, enriching and educational. As one midwife said:

It is very developing to work in new ways; you often end up in your tracks in the daily work – you do it in the same way, and it is not so much new, so it is always developing and educational to work in other ways than you are used to. (Int 7 X)

#### Home visits provide time for conversation, which promotes continuity and relationships with parents

The participants experienced that the creation of a relationship with parents was promoted during extended home visits by allocating more time for the visits and having continuity. They described the conversations that arise during the visits as their most important benefits; such conversations provided an opportunity to deepen the discussion and professionally support parents in their parenting. Home visits were experienced as less stressful than visits to a healthcare centre since the latter was accompanied by standard health controls, such as taking the mother’s blood pressure or performing other pregnancy-related controls that took time away from conversing with the parents. The participants reported that extended time can provide better quality home visits because parents are given the space to talk about what they want to discuss. As an example of this, the midwives brought a blood pressure cuff and doptone with them so they could check the mothers’ blood pressure and foetal heart sounds if they determined that it was necessary to do so. This could, for example, occur when the mother expressed concerns about the health of the foetus or when she had previously had an abnormal blood pressure. When the midwives assessed that there was no need to conduct these extra health checks, the extended home visit programme allowed them to focus on conversing with the mother instead. This is because the programme does not mandate such routine checks during home visits. The participants described how parents dared to ask more questions during home visits, sometimes posing more intimate questions they might not have asked otherwise. As one participant stated:

The families are more relaxed and they can ask about things that they might not bring up here [at the antenatal unit]. They feel comfortable that we get there […] then they can ask a little more. (Int 2 X)

Extended home visits are useful for attending to and identifying parents who feel insecure in the parental role, as well as parents with a limited social network. One participant talked about conversations with parents who felt insecure in their parenting role. After conversing with the parents during some home visits, they perceived them to be stronger and more trusting in themselves to a higher degree, as clarified in the following quotation:

The parents have said, ‘Now this works, and now we can do this…’ I think that is a positive example, that it actually helps. That they become stronger and dare to trust themselves more […]. They actually manage to have a little baby. (Int 4 Y)

The participants felt that during the extended home visits, their relationship with parents became closer and more relaxed. They describe how they came to know each other in the recurring visits and that the continuity achieved when the same individuals returned for home visits cultivated trust. The relationship with the family began at the first home visit. The participants experienced more difficulties in building a relationship with parents when continuity in the care chain could not be maintained – for example, if a different colleague made the first home visit.

#### Being humble guests in parents’ homes provides insights

When the participants were humble guests in parents’ homes, they experienced parents to be more comfortable than those attending meetings at antenatal units or the CHC. Further, home visits allowed the participants to gain insights into the home environment, which helped them better understand what kind of support the families needed. They met parents from different cultures, such that the extended home visits generated knowledge about other societies. For example, parents would describe maternity care in their previous home countries. The participants reported that parents were happy, grateful and welcoming during home visits. They believed that these parents appreciated home visits and that the visits allowed them to build trust. As one participant stated:

As long as you have a humble attitude, that we are here for you and not here to inspect […] you feel that they [parents] have become safer … you [parents] take off your armour a little. (Int 6 X)

With a humble attitude during home visits, the participants could observe how parents and children acted in their home environment. The participants experienced that they allowed parents to guide them, as opposed to visits to the maternal and CHC unit, in which the healthcare professionals are the authorities. During home visits, the participants showed interest in the parents, which resulted in the parents narrating their situation and needs. Two participants described home visits to asylum-seeking families, which, it was found, lacked the most necessary things in the home:

New arrivals, they had no furniture, and we had to arrange that as well as clothes […] and so on. It was not intended from the beginning that it would be so. (Int 1 Y)

Both midwives and CHC nurses emphasised that collaborating with a family supporter during extended home visits helped parents to obtain more detailed support and advice. The participants also had an experience in which parents questioned the purpose of the home visits during the initial home visit, which occurred during pregnancy, but changed their attitude during the visit after the participants explained how they worked. The parents then demonstrated understanding and a positive attitude towards the visits. When the participants identified parents in need of early intervention such as support and care visits beyond standard care, ethical dilemmas could arise. The participants stated that, on the one hand, they wanted to help socially disadvantaged families with early intervention and perhaps even more extra home visits, but, on the other, they did not want to reinforce parents’ sense of needing extra support and thereby reinforcing social vulnerability. The participants had to balance their desire to help the families with the families’ actual vulnerability, with the child’s best interest in mind.

#### Home visits provide the opportunity to strengthen parenting and participation in the family centre

The participants described that both parents usually participate in the first home visit. During the intervention, several participants experienced increased participation and commitment from the father/partner during visits to CHC reception and extended home visits, which they described as central to their work of strengthening gender equality. The participants explained how, in conversations with parents, they discussed the importance of both parents being involved in parenting so that they could take joint responsibility for their child from the beginning and mutually share their experiences. They strived to increase parents’ understanding of how important they both were to their child. An example of this is a father/partner who initially did not intend to attend the birth but then changed their mind after the first home visit. Several participants described the increased involvement of fathers/partners as the best part of the project. The participants experienced that they met with fathers/partners differently compared to their meetings in antenatal units or the CHC and that they could more easily involve them in conversations during home visits:

A father, he, yeah, [asked] should I be [at the Home Visit] … Then he was very involved. [He was] a little tentative at first, but then he was as active as the mother in the next visit. (Int 1 X)

During extended home visits, there is time to inform the parents about the family centre and, when needed, the participants can use an interpreter. Several participants experienced that parents who received information about family centre activities and what the open preschool offers during home visits were more involved at the family centre. These parents were more likely to participate in these activities, such as baby cafés or targeted groups, which were described as opportunities to bring parents together in the same situation. The participants experienced that parents are involved, help each other and receive support from the staff at the family centre. They also described that families who are new arrivals and newcomers are integrated into society and make new friends at the family centres. One participant described how she paid attention to lone parents:

I have been informing parents about the preschool, as we do anyway, but I think it became even clearer perhaps with the project, that you really present open preschool. So, I actually think there has been a difference, that they are more likely to go there [family centre] than they were before. (Int 1 Y)

Family supporters described how their participation in extended home visits meant that parents recognised them and asked questions when visiting the family centres. They mentioned how having their workroom close to the family centres made it easier for families to contact social services. As one family supporter said, ‘There is a lower threshold to cross’ (Int 6 X).

## Discussion

The results of this study revealed that the extended home visits implemented in collaboration between professionals with different knowledge bases serve as a foundation for providing parents with multidimensional adapted professional support. The intervention, *Enhanced Parenting—Extended Home Visits,* entails interprofessional collaboration that contributes to developing relationships between colleagues and other professionals in the project. The collaboration between the professionals enriches their work by providing a better understanding of each other’s work, and the developed collaboration facilitates contact with each other. Research has shown that collaboration is facilitated when professionals are shown respect and have a shared vision (Flood *et al*., 2019). In the antenatal and CHC context, the willingness of professionals such as midwives and CHC nurses to cooperate is facilitated if the professionals adopt a care perspective and can assist expectant and new parents throughout the whole care chain (Barimani and Hylander, 2008). The intervention in this study was inspired by the Swedish national strategy for community support and assistance to parents in their parenting (SOU, 2008: 131), which served as common ground for all professionals. One review highlighted that interprofessional collaboration must be constantly substantiated by professionals (Schot *et al*., 2020). Furthermore, Molleman *et al.* (2008) concluded that multidisciplinary teamwork appears to affect clinical and occupational autonomy and leads to an increase in professional accountability towards the multidisciplinary team. A higher level of accountability implies more feedback from team members and higher levels of self-reflection and, therefore, enhanced professionalism. Flood *et al.* (2019) described that working in a spirit of interprofessional practice implies an inner quality, an attitude or inclination that supports and enables the development of relationships that promote working together in a more connected way. Our study revealed the strengthened collaboration between professionals representing different professionals, which indicated that they developed their working methods. Subsequently, the intervention differed from standard routines since the professionals planned, performed and evaluated the home visits through interprofessional collaboration. A reflection of this is that professionals who regularly collaborate can develop in their profession, which seems to serve as a solid basis for thriving in the workplace and giving families optimal individualised support.

The participants in this study experienced extended home visits to be meaningful in that they provided time for conversation, which in turn promoted trusting relationships with parents. Clearly, extended time enhances home visits, giving parents space to talk about issues they want. According to the participants, such time represented an opportunity for the professionals to be attentive to and identify parents who felt insecure in their parental role, as well as parents with a limited social network. The issues raised were experienced as important since they provided the opportunity to deepen conversations and more professionally support parents in their parenting. The goals of the *Enhanced Parenting—Extended Home Visits* intervention were to strengthen the self-confidence of parents and to strengthen their trust in antenatal care and CHC, as well as social services. These goals seem to have been achieved with this intervention when the organisations allowed extended time for the professionals to collaborate. Another aspect that affected the professionals’ opportunities to create trusting relations with parents was continuity. The professionals described that, to create a relationship characterised as close and relaxed, the same CHC nurse and family supporter should perform each extended home visit with the family. A previous review reported that patients’ experience of trusting in relationships with care professionals depends on the main aspects of professionals’ knowledge and level of commitment in the dialogue to creating and developing the relationship (Rørtveit *et al.*, 2015). From the parents’ perspective, these extended home visits are experienced as reassuring and as promoting parental self-confidence in parenting ability, as well as giving parents a feeling of security that facilitates conversations with professionals, especially when a sense of mutual trust is created (Bäckström *et al.,* 2021). The two aspects of time and continuity seem to be important for helping professionals create relationships with parents that can stimulate parenting skills and provide children with a more secure upbringing.

In this study, the results showed that being a guest with a humble attitude provided professionals with insights into the families’ homes and allow them to observe how parents and children acted in their home environment. This, in turn, permitted conversations focused on both children’s and parents’ needs and situations, as well as involving both parents to promote equal parenting. New parents can be surprised by changes in their parental–couple relationships (Deave and Johnson, 2008), and professional support can strengthen parents’ parenting ability (Bäckström *et al.,* 2021; Larsson *et al*., 2020). Research has reported that home visits can promote healthy family functioning and positive parenting in early childhood (Minkovitz *et al*., 2016) and can especially support three-way relationships between mother and child, father and child, and mother and father, in addition to strengthening the role of each parent (Barboza *et al.*, 2018). Furthermore, research has shown that with home visits, which contribute to helping parents better understand their child’s developmental and individual needs, the effect of the early intervention can be sustained during the child’s upbringing (Aronen and Arajarvi, 2000). The intervention *Enhanced Parenting—Extended Home Visits,* which initiated extended home visits during pregnancy and continued until the children were 15 months old, entailed opportunities to provide professional support at an early stage and encourage parents’ participation in family centres’ activities. Under the CRC, it is necessary for parents and children to be given conditions that meet the unique family’s needs as well as access to healthcare professionals’ support to achieve good and equal health (CSDH, 2008).

The participants in this study narrated an ethical dilemma that could arise when they identified parents in need of early intervention. On the one hand, they wanted to help families that exhibited signs of being vulnerable or socially disadvantaged by initiating an early intervention, such as extra home visits, but, on the other, they sought to avoid unnecessarily exacerbating the family’s vulnerability. The ethical dilemma involved the participants needing to balance their desire to help with the family’s actual vulnerability, with the child’s best interest in mind. Research has shown that a lower socio-economic status can be associated with family violence (Löve *et al.*, 2021), but home visits can decrease the risk of children being exposed to parental abuse (Nygren *et al.*, 2018). From the parents’ perspective, home visits provide opportunities for professionals to investigate the child’s home environment and detect any potential danger (Bäckström *et al*., 2021). The organisation End Violence Against Children (2017) has emphasised that it is essential to engage parents in order to stop violence against children. Sweden is one of the pathfinding countries in a global partnership aimed at stopping all forms of abuse against children, in line with Goal 16.2 in Agenda 2030 and the CRC (SFS, 2018: 1197).

The *Enhanced Parenting—Extended Home Visits* project was explored from two perspectives – that of parents and that of professionals. The results from both perspectives are concordant. Parents emphasised that extended home visits as a form of professional support appear to cultivate parental self-confidence in parenting ability as well as a feeling of security that help parents contact professionals (anonymous date in manuscript). This study reported that collaboration between midwives, CHC nurses and family supporters can serve as a solid foundation on which multidimensional support can be provided to expectant and new parents. The lessons learned from this intervention are as follows: time is a prerequisite, and professionals must have sufficient administrative time to arrange collaboration with a midwife, CHC nurse and family supporter and, if needed, an interpreter. The professionals also need ample time for meetings and discussions with other professionals as well as parents in addition to arenas for such meetings. Furthermore, this study showed that professionals must have sufficient time to perform home visits quietly and with a humble attitude. It also appears that professionals must work for an organisation that permits diversity in working models and supports cooperation. To conclude, extended home visits for expectant and new parents represent a valuable form of professional support and, therefore, we have reason to recommend this type of care intervention as a future component of standard antenatal care and CHC. When implemented in this way, extended home visits can act as an investment in healthcare and social services that reduces costs for society later on, when children are growing up (Kekkonen *et al*., 2012), because it helps parents feel more secure in their parenting, in turn, improving the conditions in which children are raised.

### Strengths and limitations

The data derived from the interviews with the professionals provided a deeper understanding of the experiences of professionals working with extended home visits, which is a strength of the study. Moreover, the purposive sampling applied in the research was considered appropriate. Another strength of the study, were that all of the professionals who worked on the project providing the intervention participated in the interviews. However, only female participants were included, which could be considered a limitation in terms of both this study and the larger project. Before the interviews, an interview guide was sent to the participants. This was done with the intention of encouraging the participants to begin reflecting on their experiences providing the intervention before the interviews commenced. This could be considered a strength of the study because the participants were better prepared to answer the interview questions. At the same time, however, it could be considered a limitation because this approach may have limited the participants’ spontaneity when answering the questions. Another limitation could be that the interview questions did not encourage the participants to reflect on the different needs and experiences of various groups of parents, such as Swedish or migrant parents. No pilot interview was held before data collection, which could also be considered as a limitation. In order to evaluate the interview technique and the extent to which the generated data accomplished the aim of this study, a senior researcher read the transcripts parallel to the data collection. One of the lessons learned from collecting data for this study was that the interview technique had to differ depending on whether the interviews were held face-to-face or via telephone. The interviewers felt that they had to concentrate more on listening during the telephone interviews because they were unable to observe the participants’ facial expressions or body language. Regardless, the authors agree that both the face-to-face and telephone interviews provided rich data that effectively addressed the focus of the study. The authors represented different professionals, and they had different levels of experience using qualitative methods.

## Conclusion

The goal of the *Enhanced Parenting—Extended Home Visits* project was to strengthen parents’ confidence in their abilities, thereby building their overall self-confidence and encouraging them to develop closer relationships with staff in maternity care, CHC and social services, as well as with staff at family centres. The conclusions of this study, from the participants’ perspective, are that these goals can be achieved with this working model. Good communication skills and interprofessional cooperation towards the same goal are considered the basis on which multidimensional adapted professional support can be provided to parents. According to the results of this study, the participants developed stronger relationships with colleagues and other professionals via collaboration in the project. However, a prerequisite for such collaboration was that they were flexible with their time with parents and each other. Another goal of the project was to help parents feel more secure and willing to contact social services when needed. This was achieved by assisting parents in establishing early and positive contacts with family supporters in the intervention. The results of this study indicate that it can be easier for parents who need support from social services to contact them in this way. Another benefit is that midwives and CHC nurses experience a great advantage from the close collaboration with social services that the extended home visits project allows, and such organised cross-professional collaborations differ from how standard care is organised. The last conclusion of this study is that professionals should be able to adjust the professional support they provide based on the unique need of each parent, which would in turn demand a wide range of supportive interventions, such as extended home visits for parents.
